# Anti-aging effect of glycerophosphocholine in *Steinernema kraussei* 0657L

**DOI:** 10.3389/fphys.2024.1346579

**Published:** 2024-04-10

**Authors:** Xi-Tong Li, Xiu-Juan Qian, Hong Chen, Xing-Duo Wang, Xia Wu

**Affiliations:** Biocontrol Engineering Laboratory of Crop Diseases and Pests of Gansu Province, College of Plant Protection, Gansu Agricultural University, Lanzhou, China

**Keywords:** glycerophosphocholine, longevity, stress resistance, enzyme, activityinsulin/IGF-1 signaling pathway

## Abstract

Glycerophosphocholine (GPC) is a water-soluble small molecule found naturally in humans and foods such as milk and soybeans. It can activate the IIS pathway by regulating the expression of *daf-2*, *ins-18* and *daf-16* genes, *sek-1* and *skn-1* genes of MAPK pathway, *sod-3*, *ctl-1*, *gst-4* and other antioxidant genes. GPC can relieve symptoms related to aging in organisms. The aim of this study was to probe the effects of GPC on the longevity and stress resistance of the entomopathogenic nematode (EPN) *Steinernema kraussei* 0657L strain. The results showed that the lifespan of *S. kraussei* 0657L was significantly prolonged by 50 mM GPC treatment, which was 54.55% longer than that of the control (0 mM GPC). GPC significantly inhibited reactive oxygen species (ROS) and lipofuscin accumulation, but the body size and fecundity of *S. kraussei* 0657L had little changed. At the same time, the longevity of *S. kraussei* 0657L exposed to heat shock and UV-B radiation was significantly prolonged than that with no external stress. GPC supplementation increased the activity of antioxidant enzymes and corresponding gene expression. Under treatment with 50 mM GPC, the activities of superoxide dismutase and catalase were increased by 1.90- and 4.13-fold, respectively, the expression of the *sod-3* and *ctl-1* genes was increased by 3.60- and 0.60-fold, respectively, and harmful reactive oxygen species were removed. In addition, the expression levels of the *ins-18*, *skn-1*, *sek-1* and *gst-4* genes related to the insulin/IGF-1 signaling pathway were upregulated 1.04-, 1.84-, 2.21- and 1.24-fold, respectively. These results indicate that GPC is mainly involved in the lifespan regulation of *S. kraussei* 0657L and plays an important role in resistance to external stress by activating the insulin/IGF-1 signaling pathway and downstream PI3K/MAPK kinase, creating a new idea for improving the commercial efficacy of *S. kraussei*. It also laid a theoretical foundation for its further efficient development and utilization in the field of biological control.

## 1 Introduction

Since the first publication of *Silent Spring* by American science writer Rachel Carson in the 1960s, humans have gradually recognized the worsening ecological crisis caused by chemical overuse (Hu, 2023), the concept of biological control has been taken seriously. With the historical demand for the development of biological pest control, the method of entomopathogenic nematode (EPN) control has been applied widely from the stagnant state (Gaugler, 1990).

EPNs are globally distributed soil organisms capable of infecting and killing a vast variety of insects, and they are frequently used as biocontrol factors in insect pest management. It mainly includes *Steinernema* and *Heterorhabditis*, EPN *Steinernema kraussei* was selected in this study. Together, EPNs and pathogenic symbiotic bacteria are a lethal combination, essentially EPNs as mobile vectors for their symbiotic bacteria. EPNs seek out and invade the host insect and release the symbiotic bacteria they carry in their gut into the nutrient-rich hemolymph, where the infected insect host soon dies, the bacteria proliferate, and the nematodes feed on the bacterial and insect tissue and multiply. When host body fluids are depleted, EPNs seek out new host insects to infect. Cooperation with bacteria and the speed with which they kill pests distinguish entomopathogenic nematodes from other nematode parasites. Not all stages of EPNs are infectious, only some individuals of the 3rd juvenile stage are infectious. Under the condition of sufficient food, EPN went through the 1st, 2nd, 3rd and 4th instars, and finally developed into adults. When the food is scarce and EPN density is large, the 3rd instar larvae develop into the infective juveniles stage, that is “infective juveniles (IJs).” This is similar to the dauer of the same orders, *Caenorhabditis elegans*, which is the only free-living stage of EPNs, IJs can tolerate adversity and actively seek host insects (Dillman and Sternberg, 2012; Kin et al., 2019; Zhang et al., 2021). Although EPNs have good indoor control effects and are easily mass-produced, they are difficult to store for a long time because they are easily affected by dry, high temperature and ultraviolet radiation, which lead to the problem of the short shelf life of commercial preparations of EPNs in actual production and application (Lalitha et al., 2023). Using the free-living stage (IJs) to design high quality EPN with strong tolerance to external stress may improve its biocontrol efficacy (Maushe et al., 2023).

We generally believe that endogenous metabolites represent the physiological or pathological state of an organism and are biologically active in metabolism. Including small molecule compounds, sugars, bioactive peptides, proteins, endogenous regulatory factors and cytokines. In recent years, an increasing number of studies have shown that endogenous metabolites are related to the aging and longevity of organisms. Oleuropein and betulinic acid enhance stress resistance and extendsextend lifespan via the insulin/IGF-1 signaling pathway in *C. elegans* (Feng et al., 2021; Chen et al., 2022). Furthermore, glycogen interferes with low insulin signaling and accelerates the aging of long-lived *daf-2 C. elegans* fed a high glucose diet (Gusarov et al., 2017).

**FIGURE 1:**
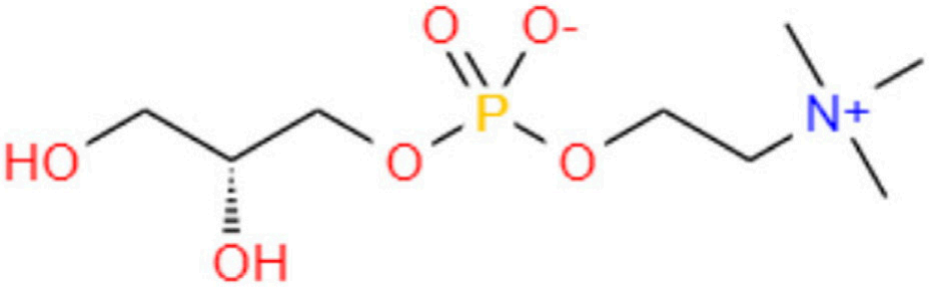
Structural formula for GPC.

GPC (C_8_H_20_NO_6_P) (Figure 1) is a choline precursor structurally similar to phosphatidylcholine (PPC), an essential nutrient needed for cell membrane integrity and signaling and lipid transport (Kim and Chung, 2021). GPC is a compound in food, such as milk, soy, liver, and sake cake, a byproduct of Japanese sake fermentation (Matsubara et al., 2018; Liu et al., 2022). Studies have shown that administration of the metabolite GPC may delay aging of the brain and potentially prevent a decline in motor function in SAMP8 mice (Matsubara et al., 2018). It is a potent nootropic for humans and is employed to combat the onset of Alzheimer’s disease and dementia and stimulate cognitive recovery, improved learning, and neurological function (Oyeneye et al., 2020). GPC significantly extends the lifespan and promotes fitness in *C. elegans* (Liu et al., 2022). Although GPC has been reported to relieve the symptoms of aging, it is unclear whether it can play a similar role in EPNs. Studies have uncovered several signaling pathways interfering with the stress response and the aging process in *C. elegans*, such as insulin/insulin-like growth factor signaling (IIS), which is conserved from worms to mammals (Feng et al., 2021). The lipid metabolism and insulin signaling pathways of nematodes are closely related to aging, and their aging characteristics (such as motor retardation and neuronal decline) are very similar to those of humans (Jin, 2022). The insulin signaling pathway is involved in metabolism and growth, and the core players in this pathway are insulin-like peptides, including insulin in mammals, IGF-I and IGF-II, phosphorylation of insulin receptor (IR) insulin receptor substrate proteins (IRS proteins) in the presence of insulin and its peptide-like proteins, and phosphorylation of insulin receptor (IR) insulin receptor substrate proteins (IRS proteins) (Partridge et al., 2011). These proteins are primarily involved in the activation of two signaling pathways: the phosphatidylinositol 3-kinase (PI3K-Akt/protein kinase B (PKB) pathway, which is responsible for most insulin metabolism; And the RAS-mitogen-activated protein kinase (MAPK) pathway, which regulates the expression of some genes and works with the PI3K pathway to control cell growth and differentiation (Taniguchi et al., 2006; Okamoto et al., 2013; Dong et al., 2023). As the same family of EPNs, *C. elegans* is mature in anti-aging research and can be used as a reference. Randy Gaugler wrote in the book Entomopathogenic Nematology that *Steinernema carpocapsae* is located in the adjacent clade of *C. elegans*, so it is obvious that *Steinernema* and *C. elegans* have similar phylogenetic relationships, and similar life and development histories, which strongly indicates that they are conserved in genes, genetic inheritance processes and pathways, then GPC metabolism should be similar in *S. kraussei* and *C. elegans.*

In this study, *S. kraussei* 0657L was implemented to explore the effects of GPC on the longevity and stress resistance of nematodes, and the mRNA levels of associated genes regulated by GPC were estimated by qRT‒PCR. The antiaging mechanism of GPC was preliminarily clarified, which will contribute to the theoretical reserve for the preparation of *S. kraussei* and field application and promote the further development of *S. kraussei* in the field of pest biological control.

## 2 Materials and methods

### 2.1 Chemicals and reagents

Glycerophosphocholine (GPC, 98% purity, Aladdin, Shanghai, China), M9 buffer (H_2_O 1000 mL, Na_2_HPO_4_ 6.0 g, KH_2_PO_4_ 3.0 g, NaCl 5.0 g, MgSO_4_·7H_2_O 0.25 g) superoxide dismutase (SOD; 5048) and catalase (CAT; 4533) assay kits were purchased from Shanghai Uplc-MS Testing Technology Co., Ltd. (Shanghai, China). H2DCFH-DA was purchased from Macklin Biochemical Co., Ltd. (Shanghai, China). All chemicals and solvents were analytical grade or higher.

### 2.2 Entomopathogenic nematode strains and maintenance

All *S. kraussei* 0657L strains isolated from Gansu Province belonging to *Steinernema kraussei* with strong resistance to low humidity stress (provided by the College of Plant Protection/Gansu Agricultural University, Biocontrol Engineering Laboratory of Crop Diseases and Pests of Gansu Province) were cultured using LB medium (the symbiotic bacterium *Xenorhabdus bovienii 0657L* isolated from *S. kraussei* 0657L was cultured in LB medium) at 25°C. Female adult *S. kraussei* 0657L was selected into a new LB medium containing *X. bovienii* 0657L by the lineation method under optical microscope. After the nematode was produced, adult *S. kraussei* 0657L was singled out and the progeny were cultured at 25°C. When *S. kraussei* 0657L grew to the same growth period J3, *S. kraussei* 0657L were washed with M_9_ buffer for further experiments. After a large number of pre-experiments in the early stage, the GPC concentration finally selected in this experiment is 0 mM, 10 mM and 50 mM, and 0 mM is treated as control (CK).

### 2.3 Lifespan assay

Synchronous J3 was picked into LB with 5 μM FUDR on Day 0 of the experiment and transferred to LB without FUDR on Day 4. After 3 days of culture, nematodes were transferred to LB with or without GPC. *S. kraussei* was transferred to new LB every 3 days, and their survival was monitored daily until all *S. kraussei* died (*S. kraussei* was considered dead if they did not respond to slight mechanical contact platinum wire). There were 5 replicates per group and 30 nematodes per replicate. The original data were analyzed by Kaplan Meier.

### 2.4 Body length and body width assay

Transferred synchronized J3 to LB with or without GPC of each group, which was recorded as Day 0 of the body length and body width assay. *S. kraussei* from Day 6 and Day 10 were randomly selected and transferred to LB in each group. *S. kraussei* was transferred to a slide with 1% sodium azide, and an anoter coverslip was gently covered. Photographs were taken under a research-grade stereomicroscope, and the segmentation tool of EP View software was used to measure the body length and width of *S. kraussei*. There were 10 replicates per group.

### 2.5 Locomotion and feeding behavior

Transferred synchronized J3 to LB with or without GPC of each group, which was recorded as Day 0 of the locomotion and feeding behavior. For the motor ability, *S. kraussei* on Day 6 and Day 10 was randomly selected and placed on blank LB medium without symbiotic bacteria. Free movement removes the symbiotic bacteria attached to the *S. kraussei* body, and the frequency of head movements of EPNs back and forth within 30 s was recorded (that is, the standard for measuring the motor ability of *S. kraussei*). From one side to the other and back (sine curve) is considered a swing. There were 10 replicates in each group, and the number of replicates was 3 per *S. kraussei*. The average value was taken as the final result.

For swallowing, the swallowing times of *S. kraussei* were recorded in 30 s. The swallowing frequency of the *S. kraussei* pharyngeal pump was observed by touching the *S. kraussei* lightly with platinum wire. There were 10 replicates in each group, and the number of replicates was 3 per *S. kraussei*. The average value was taken as the final result.

### 2.6 Lipofuscin assay

The synchronized J3 was similarly cultured in FUDR containing LB for 3 days and then transferred to normal LB on Day 4. Nematode Day 6 and Day 10 were selected to measure their lipofuscin fluorescence levels. The *S. kraussei* was transferred to a slide with 1% sodium azide, and the coverslip was covered. The intestinal lipofuscin fluorescence of *S. kraussei* was photographed using a fluorescence microscope, and the fluorescence intensity of lipofuscin was analyzed using ImageJ software, the fluorescence intensity can reflect the amount of lipofuscin deposition.

### 2.7 Reproduction assay

The synchronized J3 was transferred to LB and cultured at 25°C, and the spawning adults were transferred to a new dish every day for 4 consecutive days (spawning peak), until no more new nematodes were produced. The total number of progeny produced by each group was calculated Each group had at least 5 replicates.

### 2.8 Stress resistance assay

For heat stress, synchronized J3 was cultured at 25°C for 2 days, the temperature was raised from 25°C to 35°C, and the number of death of nematode was recorded every 2 h until all *S. kraussei* died. Under high-temperature conditions, EPNs are easily in a rigid state and need to be lightly touched with platinum wirehey were considered dead if *S. kraussei* do not react. There were five replicates per group and 30 *S. kraussei* per replicate.

For ultraviolet radiation stress, synchronized J3 was cultured at 25°C for 2 days and then exposed to 254 nm UV-B. Survival was observed and recorded every 2 h until all *S. kraussei* died. Similarly, each group had 5 replicates, and each replicate had 30 *S. kraussei*.

### 2.9 Quantification of reactive oxygen species (ROS) production

J3 was cultured in LB with or without GPC at 25°C for 5 days, and the *S. kraussei* was incubated with H2DCFH-DA for 30 min. Transferred to a slide containing 1% sodium azide. Photographs were taken using a fluorescence microscope with an excitation wavelength of 485 nm and an emission wavelength of 530 nm. The fluorescence intensity of *S. kraussei* was measured by ImageJ software.

### 2.10 Antioxidant enzyme activity determination

Synchronized J3 was cultivated with or without GPC for 2 days and exposed to 35°C for 2 h to induce the activities of SODs and CATs. Then, *S. kraussei* were collected and the attached bacteria were washed away with M9 buffer. Grinded and and centrifuged at 2,500 rpm for 10 min. The antioxidant enzyme activities in the supernatant were measured using respective assay kits. The tests were repeated in triplicate.

### 2.11 Quantitative real-time PCR (RT‒qPCR)

J3 was cultured in LB with or without GPC for 2 days, approximately 2,000 *S. kraussei* were collected in each group, their total RNA was extracted, gDNA was removed using the PrimeScript™ RT reagent Kit with gDNA Eraser, and cDNA was obtained by reverse transcription. The expression of antioxidant-related genes (*sod-3*, *ctl-2*, *ins-18*, *skn-1*, *sek-1*, *gst-4*, *daf-2*, and *daf-16*) was detected by the 2^−ΔΔct^ method, and *act-1* was regarded as an internal reference. The gene names and primer sequences for RT‒qPCR are shown in Table 1.

**TABLE 1:** Primer sequences of genes used in the experiment.

Gene	Forward primers	Reverse primers
*act-1*	5′-CTC​TTG​CCC​CAT​CAA​CCA​TGA-3′	5′-TTT​GTG​CAA​GTT​GAC​GAA​GTT​GT-3′
*sod-3*	5′-CAC​ACT​CTC​CCA​GAT​CTC​CC-3′	5′-CTC​CAA​CCA​GCG​CTG​AAA​TT-3′
*ctl-1*	5′-CGG​TCC​CTT​TTC​TAC​TCG​GA-3′	5′-AGT​CAG​GGT​GGT​ACA​GAA​GC-3′
*ins-18*	5′-TTC​GAT​GCG​CAG​GAG​AAA​AC-3′	5′-CTG​TCT​CCC​TCC​TGT​CTG​AC-3′
*skn-1*	5′-TCC​TCT​ACC​ACC​ACC​AAC​AC-3′	5′-GGC​CAT​CAT​CAC​CAA​CAT​CC-3′
*sek-1*	5′-GAG​ACG​ACA​CAC​TGA​TTG​CC-3′	5′-TTT​CAA​ACG​CAG​GTC​ACT​CG-3′
*gst-4*	5′-CTC​TTG​CTG​AGC​CAA​TCC​GT-3′	5′-TGG​CCA​AAT​GGA​GTC​GTT​GG-3′
*daf-2*	5′-TGG​ATC​TCC​ATC​GCG​AAA​CG-3′	5′-TTT​TGG​GGG​TTT​CAG​ACA​AGT-3′
*daf-16*	5′-TTA​CAT​TGC​TCG​AAG​TGC​CG-3′	5′-GGC​GAA​TCG​AGC​TCA​CGA​C-3′

### 2.12 Statistical analysis

The above experiments were repeated for more than three times. The log rank test statistical method was used to measure *S. kraussei* lifespan, and LSD and Duncan’s multiple comparison method were used to analyze the difference significance of other test data. Analysis and mapping software were used, including SPSS 19.0 (International Business Machines Corp, Armonk, New York, United States), and Origin 2021 (OriginLab Corp, Northampton, MA, United States).

## 3 Results

### 3.1 GPC leads to gains healthspan in *S. kraussei* 0657L

**FIGURE 2:**
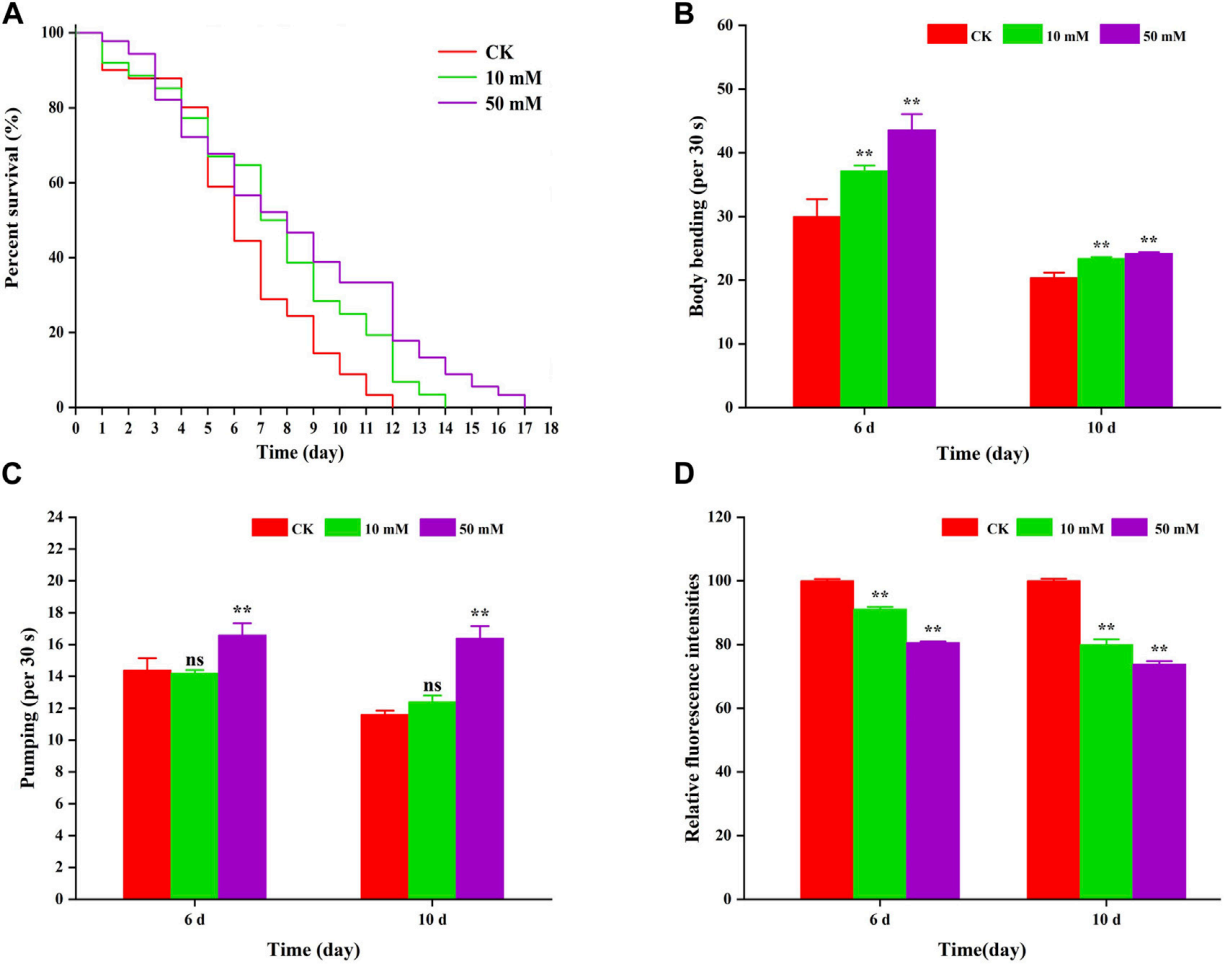
GPC partly slowed age-associated physiological decline. **(A)** Survivorship curve of *S. kraussei* 0657L treated with different doses of GPC (0, 10, 50 mM). (*n* = 30) **(B)** Age-dependent motor activity per 30 s of *S. kraussei* 0657L. **(C)** Pharyngeal pumping rates per 30 s of *S. kraussei* 0657L. **(D)** The amount of lipofuscin deposition in the gut of *S. kraussei* 0657L was measured by fluorescence intensity according to ImageJ. The long rank test statistical method was used for survival curve data. Comparisons represent one-way ANOVA followed by Student’s t-test. The average from three independent experiments was plotted, and the data are displayed as the mean ± SEM. ** *p* < 0.05; ns *p* > 0.05.

To investigate the anti-aging effect of GPC in *S. kraussei* 0657L, this study first selected one of the indexes of aging, that is lifespan for evaluation, and nematodes were fed symbiotic bacteria supplemented with GPC. Compared to the control group, supplementation with 10 mM and 50 mM GPC significantly prolonged the maximum lifespan (Figure 2A). The 10 mM GPC treatment prolonged the maximum lifespan of *S. kraussei* 0657L from 11.00 ± 0.00 days to 15.00 ± 0.00 days, which was significantly extended by 36.36% (*p* < 0.05). However, 50 mM GPC works better than 10 mM GPC, and the maximum lifespan was 17 ± 0.00 days, which was extended by 54.55% (*p* < 0.05).

Muscle often declines with the aging process, and the extent of muscle decline is reflected in motor ability. Figure 2B shows that *S. kraussei* 0657L gradually aged, and the head swing frequency decreased during the 6th to 10th day of life; however, supplementation with GPC could significantly increase the head swing frequency. On Day 6 and Day 10, after 10 mM GPC treatment, the head swing times increased from 30.00 ± 2.76 times per 30 s and 20.40 ± 0.81 times per 30 s to 37.20 ± 0.80 times per 30 s and 23.40 ± 0.24 times per 30 s, respectively, increasing by 24.00% and 14.71%. The 50 mM GPC changed the number to 43.60 ± 2.48 times per 30 s and 24.20 ± 0.20 times per 30 s, which increased by 45.33% and 18.63%, respectively.

Similarly, a decline in feeding capacity is an important criterion for aging and is often measured by the pharyngeal pump swallowing rating in nematodes. To further explore the anti-aging effect of GPC in *S. kraussei* 0657L, the swallowing ability of the pharyngeal pump was also measured after the determination of motor ability. The results also showed that compared with the control group, GPC delayed the decline in swallowing function during the aging process of *S. kraussei* 0657L (Figure 2C). Interestingly, 50 mM GPC significantly increased the swallowing frequency of *S. kraussei* 0657L, from 14.40 ± 0.75 times per 30 s to 16.60 ± 0.75 times per 30 s on Day 6 by 15.28% and from 11.60 ± 0.24 times per 30 s to 16.40 ± 0.75 times per 30 s on Day 10 by 41.38%. However, the 10 mM GPC had no significant delay effect on the decline of this function.

**FIGURE 3:**
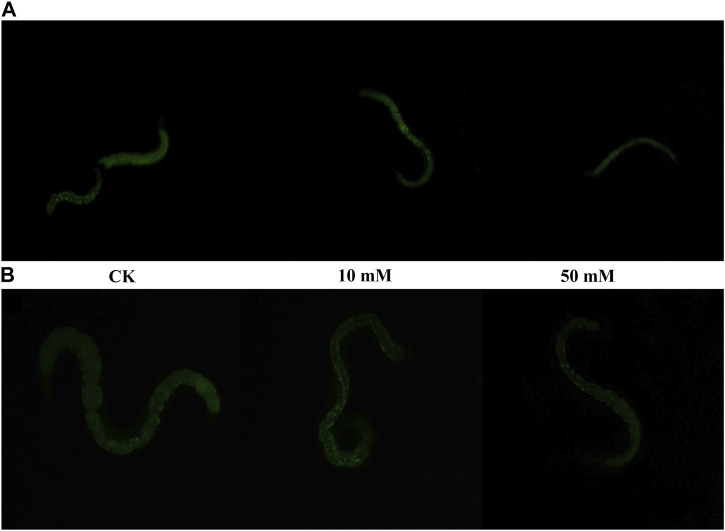
Lipofuscin-induced autofluorescence under 340–380 nm detection. **(A)** Lipofuscin fluorescence intensity in the intestine after J3 was placed on LB containing GPC for 6 days at 25°C. **(B)** Lipofuscin fluorescence intensity in the intestine after J3 was placed on LB containing GPC for 10 days at 25°C.

Nematodes contain an autofluorescent substance, lipofuscin, that accumulates during the aging process. Lipofuscin produces fluorescence gradually due to the elevated levels of oxidized proteins in lysosomes that accumulate as *S. kraussei* 0657L grows. GPC supplementation decreased lipofuscin formation (Figures 2D, 3). Compared with the control group, the 10 mM GPC group decreased lipofuscin accumulation by 8.87%, and the 50 mM GPC group decreased lipofuscin accumulation by 19.35% on Day 6. The accumulation in the 10 mM and 50 mM GPC groups decreased by 19.97% and 26.19%, respectively, on Day 10.

In summary, the above data showed that GPC significantly inhibited the accumulation of lipofuscin in *S. kraussei* 0657L, both strongly improved the decline in head mobility and pharyngeal pump swallowing ability and significantly extended their healthy lifespan.

### 3.2 GPC had no adverse effects on the body length, width and fertility of *S. kraussei* 0657L

**FIGURE 4:**
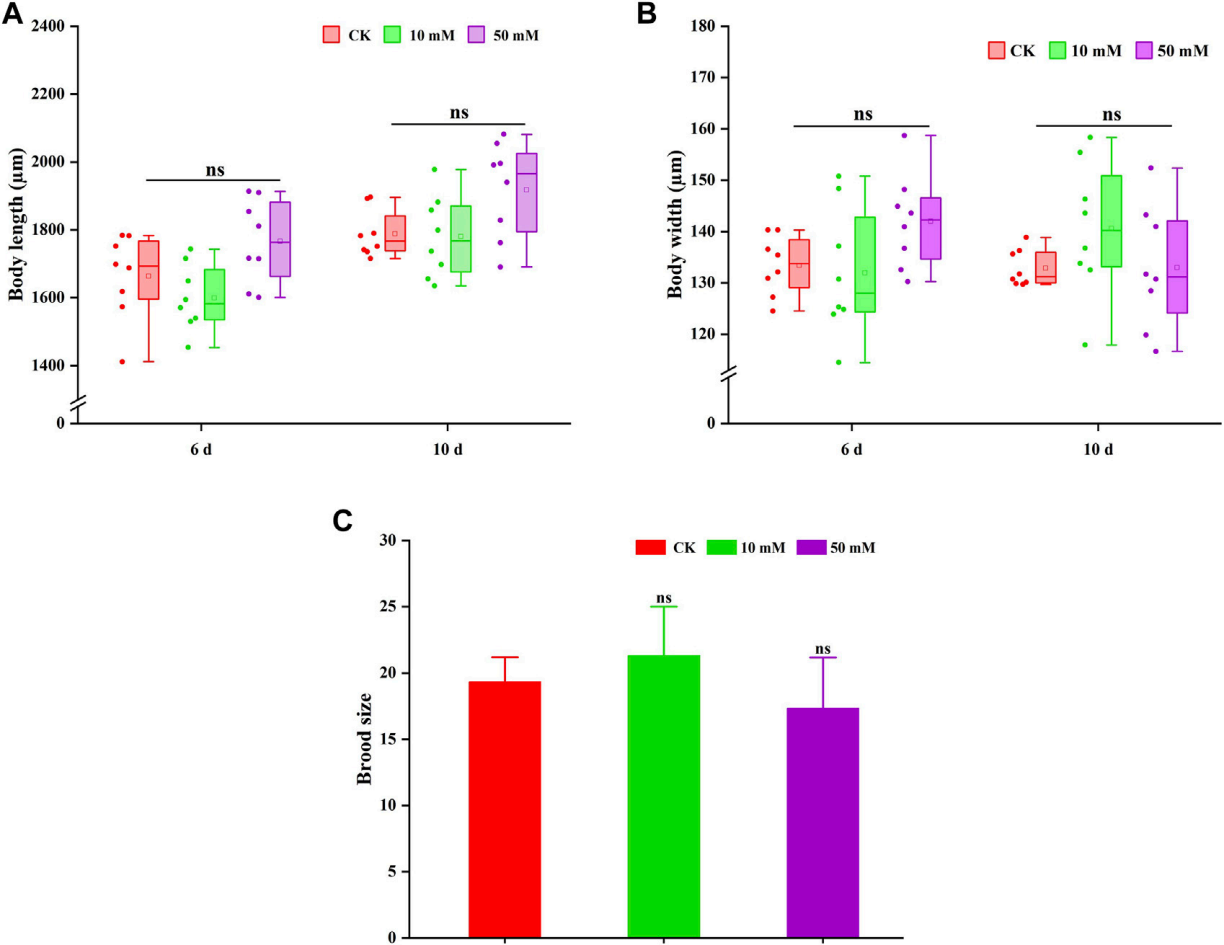
GPC has no adverse effect on the growth and development of *S. kraussei* 0657L. **(A)** Compared with the control, GPC did not change the body length in *S. kraussei* 0657L. **(B)** Compared with the control, GPC did not change the body width in *S. kraussei* 0657L. **(C)** Reproductive capacity. Comparisons represent one-way ANOVA followed by Student’s t-test. Data of **(A,B)** from ten independent and **(C)** plot data from at least three independent experiments, with the data displayed as the mean ± SEM. ** *p* < 0.05; ns *p* > 0.05.

The pro-longevity effect of most compounds on nematodes is often accompanied by the change in body size. Therefore, the changes in body length and width of nematodes treated with GPC were also measured in this study. As shown in Figures 4A, B, GPC had no significant effect on the body length and width of *S. kraussei* 0657L in comparison to the control group. These findings indicated that the lifespan extension of GPC at selected concentrations did not lead to developmental stagnation of body size.

Reproduction is usually used as an index to evaluate whether a drug is reproductively toxic to nematodes. Figure 4C shows that the 10 mM and 50 mM GPC groups did not significantly reduce the fertility of *S. kraussei* 0657L compared with the control group, which confirmed the safety of GPC.

Based on the above results, GPC did not change its body shape or inhibit the number of progeny while extending the lifespan of *S. kraussei* 0657L; that is, GPC treatments had no significant effect on some biological characteristics of *S. kraussei* 0657L.

### 3.3 GPC decreases intracellular ROS levels in *S. kraussei* 0657L

**FIGURE 5:**
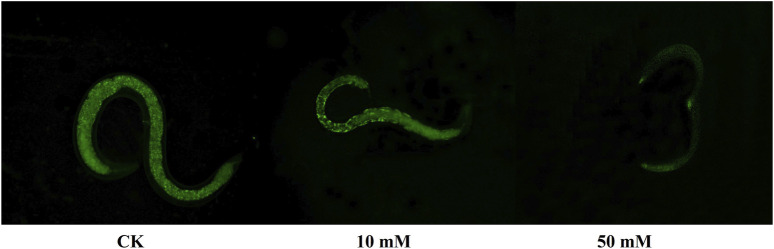
Measurement of ROS levels was achieved by H2DCFH-DA fluorescence staining.

**FIGURE 6:**
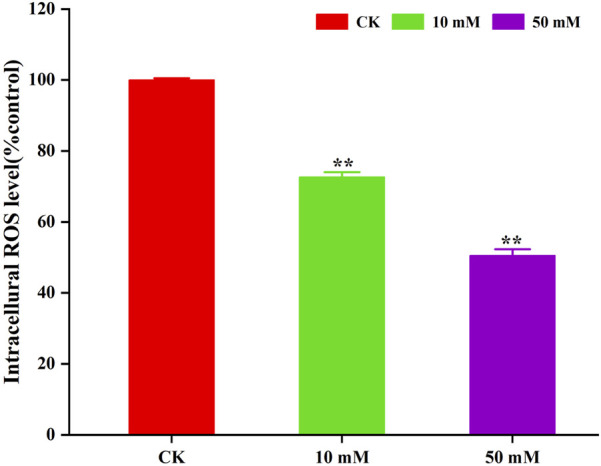
GPC decreases the intracellular ROS level in *S. kraussei* 0657L treated with 10 and 50 mM. Data from at least three independent experiments were plotted, and the data are displayed as the mean ± SEM. ** *p* < 0.05; ns *p* > 0.05.

The accumulation of ROS is considered to be one of the important causes of aging in *C. elegans* (Dong et al., 2023). The method of *C. elegans* was used for the determination of ROS levels in *S. kraussei* cells, and H_2_DCFH-DA was also used for the determination of ROS levels in *S. kraussei* cells. The fluorescence of *S. kraussei* 0657L treated with GPC was dimmer than that of the control group (Figure 5). By using ImageJ to process the images, the observed ROS levels were reduced by 27.35% and 49.75% among the GPC groups treated with concentrations of 10 and 50 mM, respectively, compared to the control group (Figure 6). The results indicated that GPC effectively inhibited reduced oxygen consumption and ROS generation.

### 3.4 GPC enhances stress resistance in *S. kraussei* 0657L

**FIGURE 7:**
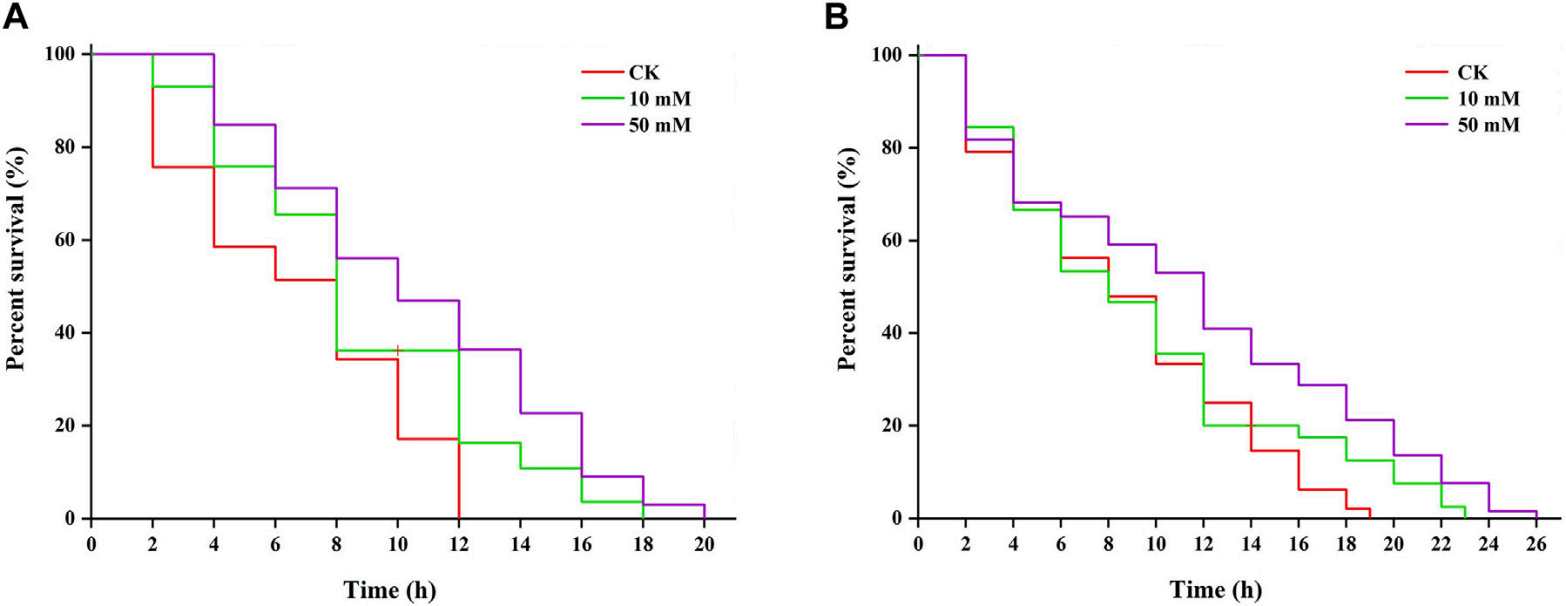
GPC enhances stress resistance in *S. kraussei* 0657L. **(A)** GPC increased the mean lifespan of *S. kraussei* 0657L under heat (38°C) (*n* = 30). **(B)** GPC increased the mean lifespan of *S. kraussei* 0657L under UV-B (280–315 nm) stress (*n* = 30). The survival curve data were analyzed by the long rank test.

Research shows that increased longevity is correlated with tolerance to stresses (Maushe et al., 2023). The results of this study showed that *S. kraussei* 0657L treated with GPC could increase resistance to heat and UV-B radiation stresses. Under 38°C-induced heat stress, 50 mM GPC pretreatment provided significant (*p* < 0.05) protection and prolonged lifespan by 61.66% compared with the control group, and the maximum lifespan increased from 12.00 ± 0.00 h to 19.33 ± 0.67 h (Figure 7A). Under UV-B-induced heat stress, compared with the control group, the maximum lifespan of *S. kraussei* 0657L in the 50 mM GPC pretreatment group was significantly (*p* < 0.05) extended by 32.14%, from 17.33 ± 2.31 h in the control group to 24.67 ± 1.54 h (Figure 7B). These results showed that GPC could improve the health lifespan of *S. kraussei* 0657L and increase its anti-stress ability.

### 3.5 GPC enhanced antioxidant enzyme activities in *S. kraussei* 0657L

**FIGURE 8:**
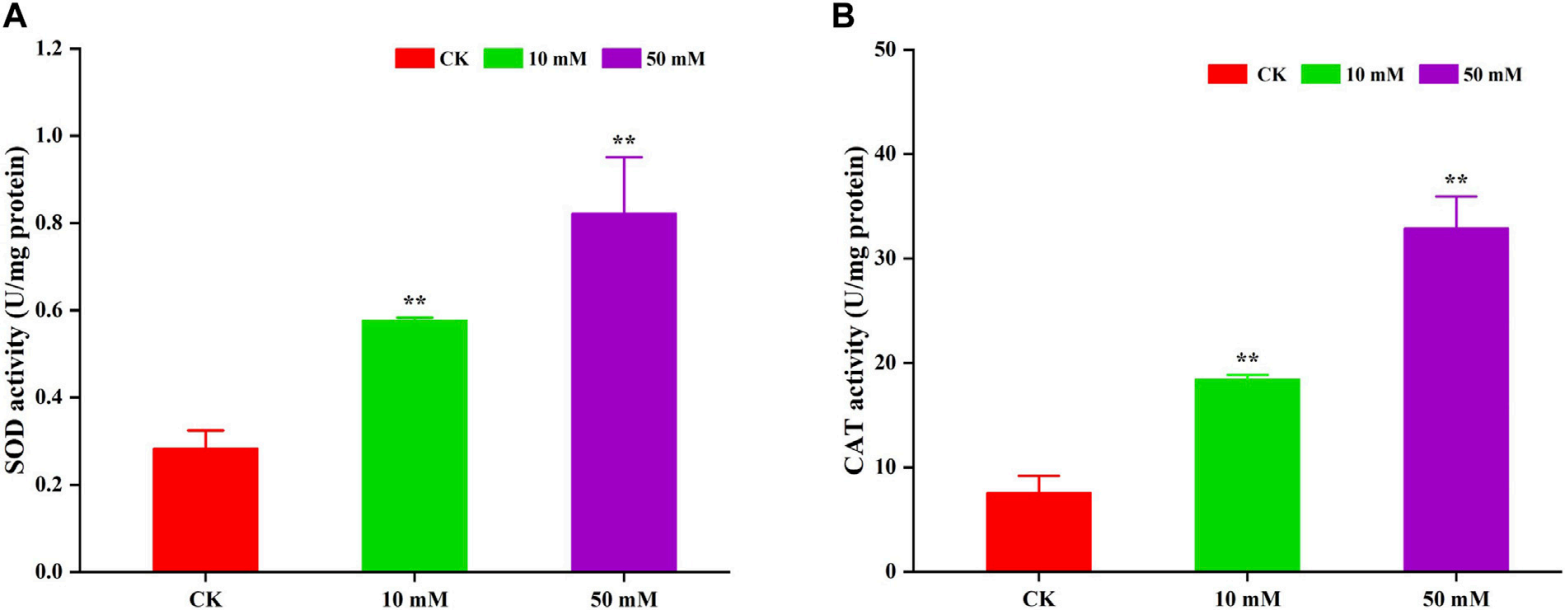
GPC enhanced antioxidant enzyme activities in *S. kraussei* 0657L. **(A)** SOD activity was increased by 10 and 50 mM GPC compared with the control. **(B)** CAT SOD activity was increased by 10 and 50 mM GPC compared with the control. Data from at least three independent experiments were plotted, and the data are displayed as the mean ± SEM. ** *p* < 0.05; ns *p* > 0.05.

Antioxidant enzymes can scavenge superoxide radicals that cause oxidative damage to biomolecules and are mainly used to alleviate oxidative damage. In this study, the activities of SOD and CAT were determined. The results are shown in Figure 8. GPC could significantly (*p* < 0.05) increase the activity of SOD and CAT in *S. kraussei* 0657L. For SOD, compared to the control group, 10 and 50 mM GPC increased its activity by 1.04- and 1.90-fold. The CAT activity of the 10 and 50 mM GPC treatment groups was 2.23 times and 4.13 times that of the control group, respectively. The above data indicate that the antioxidant enzymes SOD and CAT can activate the oxidative defense system, thereby reducing oxidative damage.

### 3.6 GPC treatment influenced mRNA expression in *S. kraussei* 0657L

**FIGURE 9:**
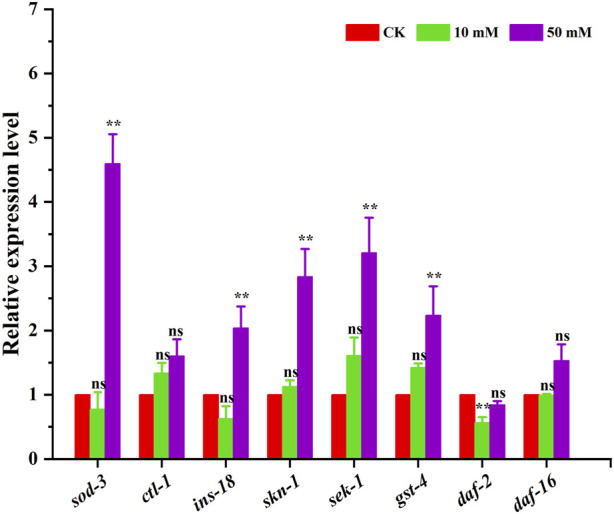
RT‒qPCR detection of the expression of antioxidant genes. The gene transcription level was normalized to the internal control gene *act-1*. ** *p* < 0.05; ns *p* > 0.05.

The above results indicated that GPC can effectively retard aging in *S. kraussei* 0657L, but the exact mechanism of action is not clear. In this study, the insulin/IGF-1 signaling pathway-related genes *sod-3*, *ctl-1*, *ins-18*, *skn-1*, *sek-1*, *gst-4*, *daf-2* and *daf-16* were selected to further explore the possible mechanism at the transcriptional level. The RT‒qPCR results showed that compared with the control group, which was upregulated by 3.60-, 1.04-, 1.84-, 2.21- and 1.24-fold, respectively, 50 mM GPC treatment mainly improved the expression of the *sod-3*, *ins-18*, *skn-1*, *sek-1* and *gst-4* genes and downregulated the expression of the *daf-2* gene by 0.16-fold but had no significant effect on *ctl-1* and *daf-16* (Figure 9).

## 4 Discussion

EPNs are promising biological control agents for numerous agricultural pests. However, the complex environment that nematodes face in controlling pests in the field hinders their use. From mass propagation preparation, storage and transportation to field application, the main reason for the reduced control efficiency is the shortening of the shelf life of nematodes due to various environmental stresses in this process. Based on longevity studies of *C. elegans*, we hypothesized that drug interference, in particular bioactive natural compounds, could regulate the longevity signaling pathway to extend median lifespan and maximum healthspan and enhance anti-stress ability in EPNs (Feng et al., 2021). Therefore, we regard the EPN *S. kraussei* 0657L as a research object to study the effects of the water soluble small molecule substance GPC on their lifespan and motor ability. Our study shows that certain concentration of GPC can extend the lifespan of *S. kraussei* 0657L without dietary restriction (DR) and delay the age-related decline in physical motor capacity. Moreover, the accumulation of lipofuscin was also significantly reduced, which produces self-fluorescence in *S. kraussei* 0657L.

Metabolism is closely related to aging. The endogenous metabolite GPC is a natural source of choline, and reports show that GPC declines in plasma with age, indicating its possible role in longevity regulation (Liu et al., 2022). GPC reportedly prevents aging-related decline in cognitive function (Narukawa et al., 2020) and has been found to promote longevity and fitness in the model organism *C. elegans* during aging (Liu et al., 2022). In addition, there are other metabolites that also show anti-aging activity in organisms, such as alpha-ketoglutarate, a key metabolite in the TCA cycle, which has longevity effects (Shahmirzadi et al., 2020). The maintenance of efficient DNA repair by NAD^+^ recharging in cells, *C. elegans*, and mice may delay the onset of aging (Zhou et al., 2020), and increased N-glycan precursor synthesis in the hexosamine pathway improves endoplasmic reticulum protein homeostasis and prolongs the lifespan of *C. elegans* (Denzel et al., 2014). The involvement of endogenous metabolites in human aging regulation has been widely acknowledged recently. It is regarded as an exogenous compound when supplementing GPC for EPNs, although it is a natural product. Thus, the interference of most exogenous substances may cause adverse reactions for organisms, such as changes in body length and width and a decline in fertility. In this study, we found that GPC supplementation did not cause significant changes in the body size and reproductive capacity of *S. kraussei* 0657L, meaning that the right concentration of GPC supplementation had no negative effects on the growth and development of *S. kraussei* 0657L.

The mitochondrial free radical theory of aging (MFRTA) is one of the most important theories of aging and proposes that aging is caused by the destruction of macromolecules by mitochondrial ROS. Therefore, ROS accumulation is considered to be one of the important causes of senescence, and decreasing ROS production can effectively delay aging (Dong et al., 2023). Normally, with increased age, ROS cause oxidative damage to DNA, proteins and lipids, ROS levels rise, damage accumulates, cell dysfunction occurs, and the aging process accelerates, which leads to a shortened lifespan (Qi et al., 2021). In this study, after supplementing *S. kraussei* 0657L with GPC in concentrations of 10 mM and 50 mM, ROS levels decreased significantly in *S. kraussei* 0657L, indicating that certain concentration of GPC cleared harmful ROS. The clearance of reactive oxygen species depends on the action of antioxidant enzymes in the antioxidant defense system (Chen et al., 2023). SOD catalyzes the disproportionation of O^2−^ to produce O_2_ and H_2_O_2_, and CAT promotes the decomposition of H_2_O_2_ into H_2_O and O_2_ (Zhu et al., 2023). In this work, our assay results show that supplementation with that the right concentration of GPC can increase SOD and CAT activity, which helps to remove free radicals and protect cells from oxidative damage, thus resisting aging caused by oxidative stress.

A large body of credible evidence suggests that longevity is often accompanied by increased resistance to environmental stress, and stress resistance might be a determining factor in lifespan (Lithgow and Walker, 2002; Lin et al., 2020). In this experiment, we also confirmed that GPC treatment prolonged the lifespan of *S. krausse*i 0657L subjected to heat stress and UV-B stress and improved its tolerance. This result suggested that appropriate concentrations of GPC had the potential to help *S. kraussei* 0657L resist stress. And consistent with the effect of GPC in *C. elegans* (Liu et al., 2022), with the increase of the concentration, the longer the lifespan of *S. krausse*i, the stronger the stress resistance, that is, GPC also has a concentration-dependent effect on the longevity and stress resistance in *S. krausse*i.

**FIGURE 10:**
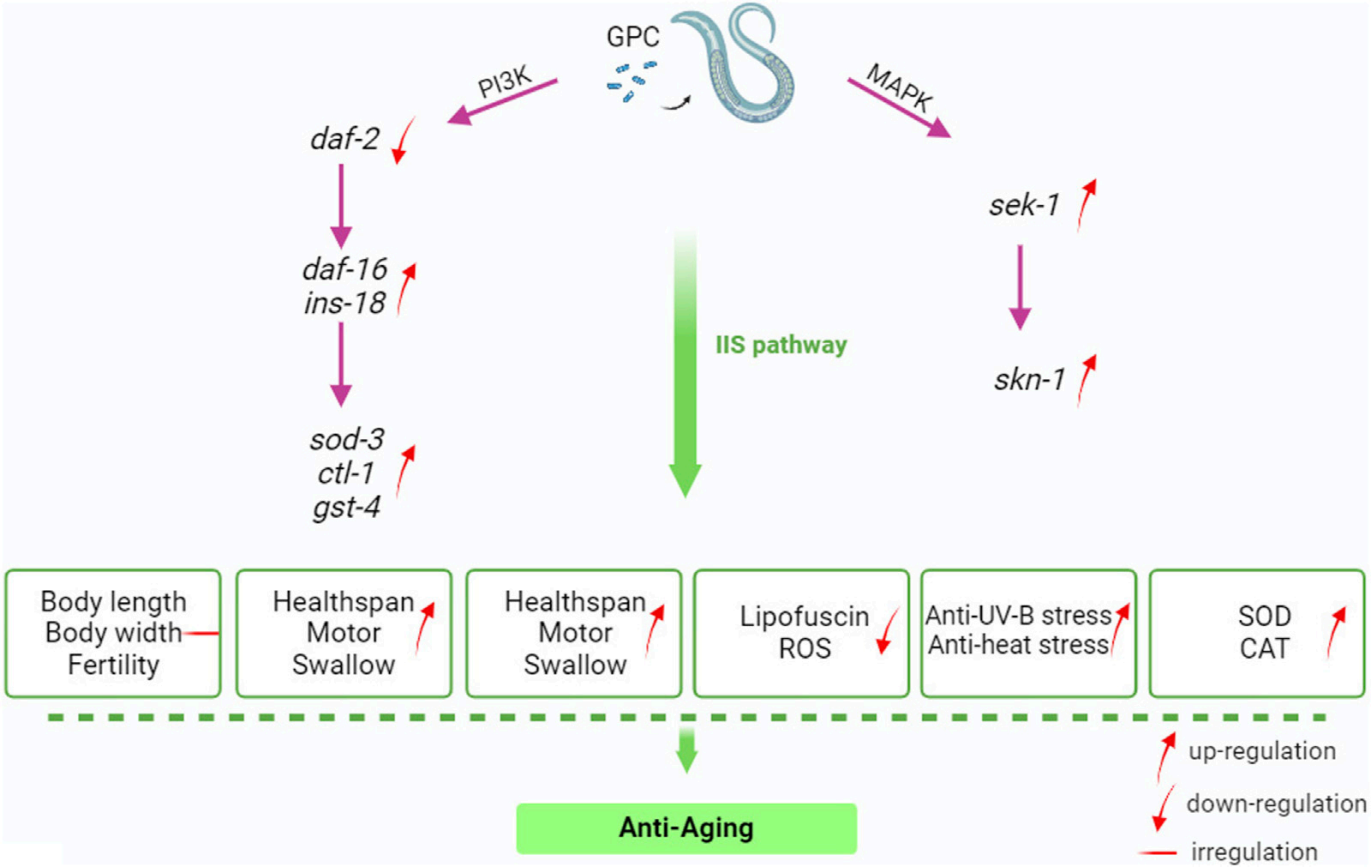
Mechanism by GPC prolongs the lifespan of *S. kraussei* 0657L.

IIS about nutrient availability, number of or competitors, and temperature; these variables may be interpreted as predictors of future survival and reproductive conditions (Zheng et al., 2017). Much of the *C. elegans* systemic response to these environmental factors is mediated through IIS and its interactions with other signaling pathways (Murphy and Hu, 2018). IIS is involved in metabolism and growth and is highly conserved in evolution from invertebrates to mammals (Partridge et al., 2011). Given this, we selected several antioxidant-related genes in *S. kraussei* 0657L based on longevity studies of *C. elegans* for RT‒qPCR assays and found that the main genes that could prolong the lifespan of *S. krausse*i 0657L and resist stress were *sod-3*, *sek-1*, *skn-1*, *gst-4*, and *ins-18*. GPC supplementation activates phosphoinositol 3 kinase AGE-1/PI3K, which triggers downstream phosphorylation from 3-phosphoinositol dependent kinase (PDK-1) to AKT/protein kinase B family members (AKT-2, AKT-1, and SGK-1). INS-18 plays a role in longevity by inhibiting DAF-2 receptor signaling, and phosphorylation of upstream AKT-1 and DAF-2 regulates the expression of downstream gene *daf-16*, thereby enhancing the regulation of downstream target genes, and upregulates the expression of extracellular superoxide dismutase gene *sod-3*, choline transporter-like protein-encoding gene *ctl-1* and glutathione mercaptotransferase gene *gst-4*. In the MAPK kinase cascade reaction, the double-specific mitogen-activated protein kinase gene *sek-1* in the MAP2K kinase pathway acts through the alkaline leucine zipper transcription factor SKN-1, which improves the stress resistance in *S. kraussei* 0657L and prolongs their lifespan accordingly (Figure 10). This explains why the insulin/IGF-1 signaling pathway is the key pathway by which GPC affects lifespan in *S. kraussei* 0657L. The downstream pathway of IIS pathway mainly includes PI3K-Akt and MAPK, MAPK is also a component of life regulation. The results of this study showed that GPC activated the expression of *daf-16* gene in IIS pathway, *sek-1* and *skn-1* genes in MAPK pathway, and antioxidant genes such as *sod-3*, *ctl-1*, *ins-18* and *gst-4*. The upregulation of *daf-16* combined with the downregulation of *daf-2* and the activation of the downstream target antioxidant genes *sod-3*, *ctl-1*, *ins-18* and *gst-4* regulated the longevity of *S. kraussei* 0657L. In addition, in the MAPK pathway, the expression of *skn-1* and *sek-1* was upregulated after GPC treatment, especially the expression of *sek-1*. The activation of these two genes also provides a basis for the study of the MAPK cascade downstream of insulin signaling pathway in *S. kraussei*.

## 5 Conclusion

The antioxidative function in *S. kraussei* 0657L was pilot studied in this paper, and the results showed that GPC could inhibit the deposition of lipofuscin and reduce ROS levels in *S. kraussei* 0657L. The activity of SOD and CAT enzymes and the resistance of *S. kraussei* 0657L to heat stress and UV-B stress were improved significantly, and it also had no adverse effects on growth and development, reproductive or motor ability while exerting anti-aging activity. The molecular mechanism of the anti-aging activity of GPC was further explored, and it was found that it upregulated antioxidant gene transcription and promoted antioxidant capacity and lifespan in *S. kraussei* 0657L. This effect of GPC was associated with the IIS pathway and depended upon DAF-16 as well as SEK-1. This experiment provides a theoretical basis and practical basis for the commercial production and practical application of *S. kraussei* 0657L. For the application of the results of this study, the appropriate concentration of GPC should be added to J3 to improve its stress resistance during the stage of large-scale propagation of *S. kraussei*, and the shelflife of *S. kraussei* preparation can be enhanced under poor transport conditions and inappropriate preservation conditions after the *S. kraussei* 0657L preparation is synthesized.

## Data Availability

The original contributions presented in the study are included in the article/supplementary material, further inquiries can be directed to the corresponding author.
